# Endoscopic submucosal dissection of early gastric angle cancer by using a simplified robot-assisted device for traction

**DOI:** 10.1055/a-2227-6465

**Published:** 2024-01-17

**Authors:** Can Cui, Xuefeng Lu, Xiu-Li Zuo, Rui Ji

**Affiliations:** 1 91623 Department of Gastroenterology, Qilu Hospital of Shandong University, Jinan, Shandong, China; 2 91623 Shandong Provincial Clinical Research Center for digestive disease, Shandong, China; 3 91623 Laboratory of Translational Gastroenterology, Qilu Hospital of Shandong University, Jinan, Shandong, China; 4 91623 Robot engineering laboratory for precise diagnosis and therapy of GI tumor, Qilu Hospital of Shandong University, Jinan, Shandong, China

Endoscopic submucosal dissection (ESD) is the standard technique for resection of gastric superficial tumors. However, in difficult locations of the stomach, a vertical approach toward the muscular layer is always unavoidable and results in a higher risk of damage 1 . To help address these issues, we designed a flexible auxiliary single-arm transluminal endoscopic robot (FASTER). The operator can utilize its external control system to precisely manipulate the grasping forceps, exerting a controlled pulling force in multiple directions away from the gastric wall ( Fig. 1 ) 2 .

**Fig. 1:**
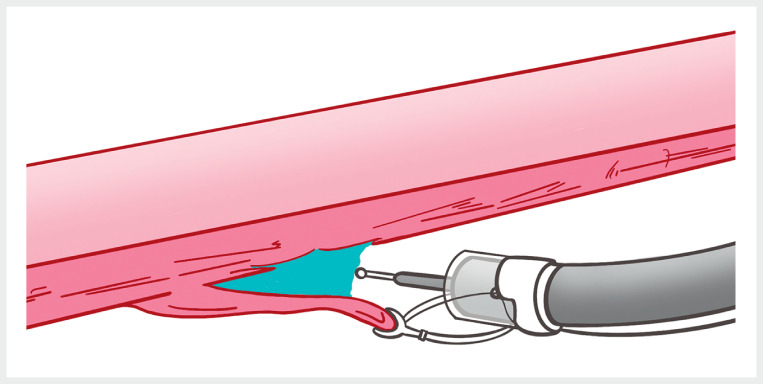
Forceps grasped the mucosa and provided a downward pulling force.

In this case, we completed a successful gastric angle ESD with the assistance of FASTER ( Video 1 ). Firstly, a flat lesion was observed at the gastric angle ( Fig. 2 ). Then, lesion marking, submucosal injection, and circumferential mucosal incision were performed. Next, the robot arm was attached to the tip of the conventional endoscope by a soft hood. The forceps were fixed at the six oʼclock position to grasp the lesion edge, providing downward traction. The operation progressed with an antegrade approach from the angle to the antrum. During the process, the submucosa was completely exposed and blood vessels were clearly visible ( Fig. 3 , Fig. 4 ). The position of the forceps could also be changed to achieve multi-position traction. Finally, the lesion was successfully completely removed ( Fig. 5 ). The total duration of the submucosal dissection was about 33 minutes. There were no complications during the operation.

Endoscopic submucosal dissection of early gastric angle cancer by using a simplified robot-assisted device for traction.Video 1

**Fig. 2:**
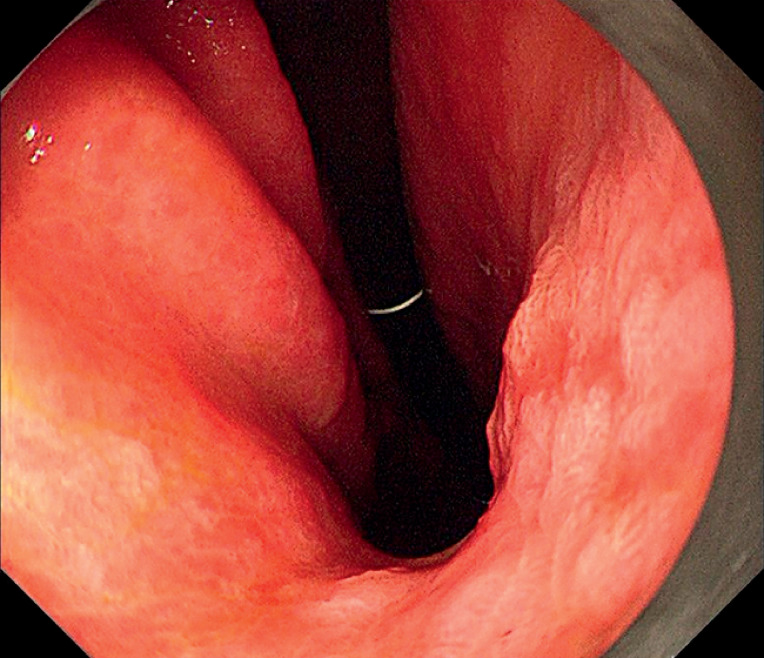
A flat lesion (IIc, 40 mm in diameter) was observed at the gastric angle.

**Fig. 3:**
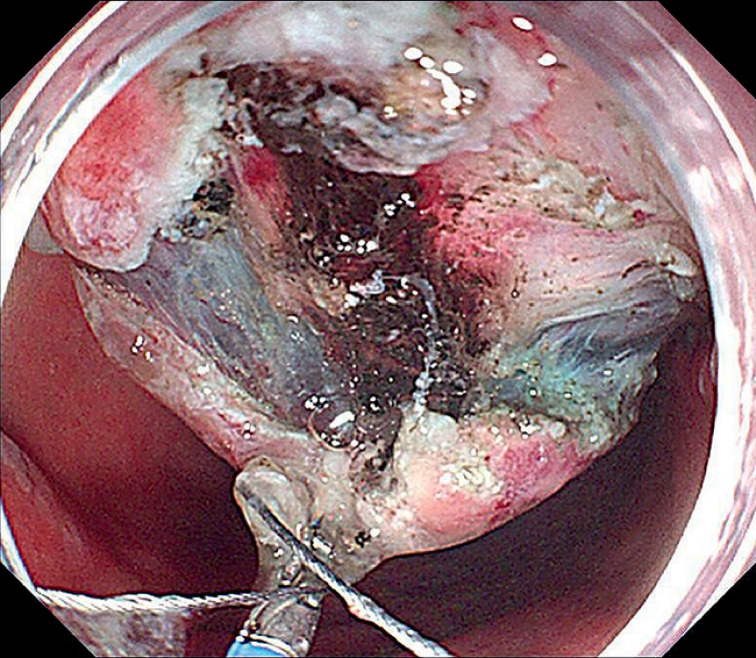
With robot-assisted traction, the submucosa was completely exposed.

**Fig. 4:**
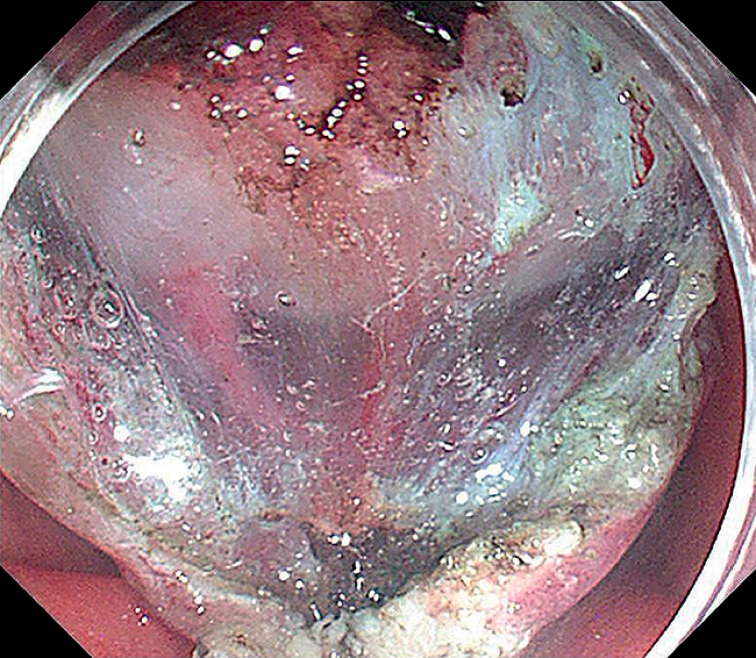
The blood vessels were clearly visible.

**Fig. 5:**
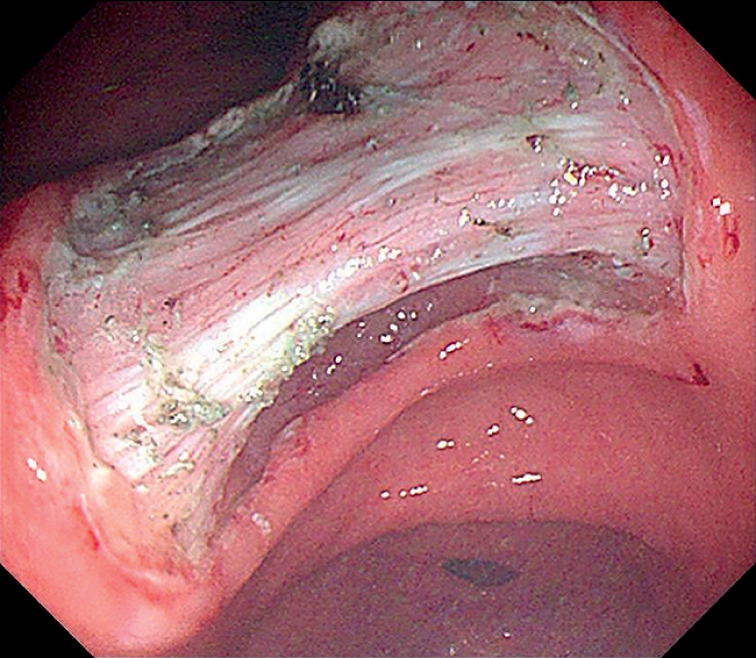
Condition after the lesion was completely removed.

In our experience, we believe that FASTER has three representative advantages: (1) because of its flexibility, traction in both multi-position and multi-angle can be achieved; (2) it enabled an antegrade, tangential approach; and (3) it can effectively cope with difficult gastric angle lesions. It is not difficult to see that FASTER makes ESD easier and safer and provides a new strategy for gastric ESD. More cases and longer follow-up are needed to validate the advantage of this technique.

Endoscopy_UCTN_Code_TTT_1AO_2AG

## Notes

Endoscopy E-Videos https://eref.thieme.de/e-videos
*E-Videos* is an open access online section of the journal *Endoscopy* , reporting on interesting cases and new techniques in gastroenterological endoscopy. All papers include a high-quality video and are published with a Creative Commons CC-BY license. Endoscopy E-Videos qualify for HINARI discounts and waivers and eligibility is automatically checked during the submission process. We grant 100% waivers to articles whose corresponding authors are based in Group A countries and 50% waivers to those who are based in Group B countries as classified by Research4Life (see: https://www.research4life.org/access/eligibility/ ). This section has its own submission website at https://mc.manuscriptcentral.com/e-videos .
